# Novel Compound Polysaccharides from Chinese Herbal Medicines: Purification, Characterization, and Antioxidant Activities

**DOI:** 10.1155/2022/9973419

**Published:** 2022-06-10

**Authors:** Xu Yang, Haiyu Ji, Yingying Feng, Juan Yu, Anjun Liu

**Affiliations:** ^1^Tianjin Institute for Food Safety Inspection Technology, Tianjin 300308, China; ^2^Key Laboratory of Food Nutrition and Safety, Ministry of Education, School of Food Science and Engineering, Tianjin University of Science and Technology, Tianjin 300457, China

## Abstract

The present study investigated physicochemical properties and antioxidant activities *in vivo* and *in vitro* of purified compound polysaccharides (CPs-1) from Chinese herbal medicines, composed of lotus leaf, hawthorn, Fagopyrum tataricum, Lycium barbarum, Semen cassiae, and Poria cocos with the mass ratio of 2 : 4 : 2 : 1 : 1.5 : 1. The HPGPC profile and FT-IR spectra indicated that the average molecular weight of CPs-1 was 38.7 kDa and possessed the *α*- and *β*-D-pyranose, respectively. The methylation analysis and NMR spectrum demonstrated that CPs-1 had a →6)-*β*-D-Glcp-(1→6)-*β*-D-Glcp(1→ backbone. Furthermore, the antioxidant assays *in vitro* revealed that CPs-1 displayed high scavenging abilities for DPPH, hydroxyl, and reducing power, as well as ABTS and superoxide scavenging capacity. The antioxidant experiments *in vivo* revealed that CPs-1 could significantly enhance CAT, SOD, and GSH-Px activities and dramatically reduce MDA levels in liver and serum of high-fat mice. Therefore, CPs-1 could be potentially incorporated into pharmaceutical products or functional foods as a natural antioxidant.

## 1. Introduction

Free radicals are the generic terms of atoms, atomic groups, or molecules in a particular state containing unpaired electrons generated during biochemical reactions in the body [1]. Reactive oxygen species (ROS) are produced in the general physiological metabolism of human cells and tissues, significant in maintaining oxidative balance in antioxidant defense mechanisms [2]. However, excessive free radicals, especially ROS, lead to oxidative stress relevant to diabetes [3], Alzheimer [4] cardiovascular disease [5], nephrosis [6], cancer [7], arteriosclerosis [8], and many other aging-related disorders [9]. Antioxidants can protect the body against oxidative damage and delay chronic disease pathogenesis to a certain extent via restraining and scavenging ROS [10, 11]. Due to their antioxidant activity (one of the most important bioactivities of polysaccharides), polysaccharides from Chinese herbal medicines (CHMs) have been broadly used as safe, stable, and effective natural antioxidants, because they possess less toxicity and side effects, compared with synthetic antioxidants [12, 13]. As a result, developing new multifarious CHM-derived polysaccharides has received considerable interest.

As CHMs, lotus leaf, hawthorn, *Semen cassiae*, *Fagopyrum tataricum*, *Poria cocos*, and *Lycium barbarum* are edible plants with medicinal benefits (called “medicine food homology” in China) [14]. Many studies have been published on the characterization and bioactivities of polysaccharides obtained from the above CHMs [15–18]. The compound polysaccharides (CPs) are complex with two or two more polysaccharides. It has been reported to show multiple pharmacological activities including antiviral, anti-inflammatory, antioxidant, and radioresistant and immunomodulating properties [19–22]. Furthermore, CPs prevent rats from obesity and related metabolic issues [23]. Moreover, CPs own higher radical scavenging activity than that of the single polysaccharide [24]. The compound CHM ingredients can better enhance immunity than the single CHM ingredient [25]. The characterization and antioxidant activities of crude CPs from the above six CHMs have been investigated in our published article [26]. However, the purification, structure, and antioxidant activities of polysaccharides from the aforesaid crude CPs still remain unknown.

The current study designated to identify the characterization and antioxidant activities of purified CPs. Firstly, crude CPs were isolated with Sephadex G-75 column, and the preliminary structure was determined by methylation and nuclear magnetic resonance (NMR), high-performance gel permeation chromatography (HPGPC), chemical analysis, ion chromatography (IC), UV spectrum, and Fourier transform-infrared spectroscopy (FT-IR). Furthermore, *in vitro* and *in vivo* antioxidant activities of CPs were investigated based on their capacities to scavenge radical (DHHP, ABTS, hydroxyl, and superoxide anion) and reducing powers, as well as the murine serum and liver concentrations of catalase (CAT), glutathione peroxidase (GSH-Px), malondialdehyde (MDA), and superoxide dismutase (SOD).

## 2. Materials and Methods

### 2.1. Materials and Reagents

The fruits of *Poria cocos* (Schw.) Wolf, fruits of *Lycium barbarum* L., lotus leaves of *Nelumbo nucifera* Gaertn., seeds of *Cassia tora* Linn. (Semen cassiae), fruits of *Fagopyrum tataricum* (L.) Gaertn., and *Crataegus pinnatifida* (hawthorn) were obtained from Bozhou Shenghao Biotechnology Co., Ltd. (Anhui, China). Samples were gently processed for storage at 4°C. The dextran standards were purchased from Pharmacia Co. (New York, USA). Sephadex G-75 was gained from GE Healthcare Life Science (Piscataway, NJ, USA). The monosaccharide standards were purchased from Sigma-Aldrich Chemical Co. (St. Louis, USA). The assay kits of SOD, CAT, MDA, and GSH-Px were acquired from Nanjing Jiancheng Bioengineering Institute (Nanjing, China). Other chemicals or reagents were of analytical grade.

### 2.2. Extraction and Purification of CPs

The lotus leaf, hawthorn, Fagopyrum tataricum, Lycium barbarum, Semen cassiae, and Poria cocos were powdered using a grounding equipment (Model 800C, Yongkang Aizela Electric Appliance Co., Ltd., Zhejiang, China), respectively. Then, the fine powder was mixed at the mass ratio of 2 : 4 : 2 : 1 : 1.5 : 1 [27, 28]. According to our preliminary experiments [26], 10 g of the compound was extracted with deionized water (30 mL/g, 65°C, 45 min) three times using the ultrasonic-assistant extraction. After vacuum filtration, the aqueous extracts were mixed with 2% pectinase, at 50°C for 2 h, followed by a 5 min incubation at 95°C for enzyme inactivation. Then, a rotary evaporator was initially employed to remove excessive water from the mixture (SY-2000, Yarong Technology and Science Inc., Shanghai, China) at 55°C with the presence of vacuum, followed by mixing with ethyl alcohol (1 : 4 dilution ratio of concentrated mixture and ethyl alcohol). The diluted sample was stored overnight at 4°C. Then, the mixture was 15 min centrifuged at a speed of 4500 r/min. The pellets were three-time washed with dehydrated ethanol. After redissolving of collected precipitate (termed crude CPs), dialysis and the lyophilization were subsequently conducted at -50°C under vacuum. Finally, the obtained solid was ground to powder.

CPs were dissolved in deionized water to reach 10 mg/mL, followed by separating into different fractions based on molecular weights (MWs) via ultrafiltration centrifugation with a 10 kDa MWCO (Sartorius, Göttingen, Germany) (at 4000 rpm for 20 min). Two fractions were obtained, i.e., CPs-1 (>10 kDa) and CPs-2 (1-10 kDa). The preexperiments revealed that CPs-1 presented stronger antioxidant activities than CPs-2. CPs-1 was purified using a permeation column (Sephadex G-75 gel, 4.5 × 60 cm) to acquire homogeneous polysaccharides. CPs-1 was eluted and collected using 5 mL deionized water at 1.0 mL/min flow rate. Total carbohydrate was determined by phenol-sulfuric acid. The protein in CPs-1 was examined by bovine serum albumin and Coomassie Brilliant Blue G-250.

### 2.3. Characterization of CPs-1

#### 2.3.1. Analysis of Molecular Weight Distribution

The molecular weight distribution of CPs-1 was determined by HPGPC based on previously published method [29]. The calculation of the molecular weight of CPs-1 was based on the dextran standards.

#### 2.3.2. Analysis of Monosaccharide Composition

The monosaccharide composition and molar ratios of CPs-1 were obtained using Dionex ICS-5000 chromatographic system (CA, USA). An aliquot of 5 mg CPs-1 sample was hydrolyzed in 1.0 mL trifluoroacetic acid (TFA, 2.0 mol/L) at 110°C for 4 h in a sealed tube. The recovery of TFA was achieved by adding methanol in a N_2_ atmosphere. The 100 times dilution of the dried hydrolysate was obtained using 1.0 mL of deionized water. The elution was completed using NaAC (200 mM) and NaOH (10 mM) solutions at 0.5 mL/min flow rate at a column temperature of 30°C to generate 25 *μ*L sample [30].

#### 2.3.3. FT-IR and UV Spectrometric Analyses

Potassium bromide (KBr) was added into the CPs-1, and subsequently the mixture was pressed into a tablet. The resulting signal of FTIR spectra analysis at the detector (VECTOR-22 IR, Bruker, Karlsruhe, Germany) presents as a spectrum from 4000 cm^−1^ to 400 cm^−1^. A UV-2500PC spectrophotometer (Shimadzu, Kyoto, Japan) in 200-400 nm was utilized to document the UV spectra of CPs-1 [31].

#### 2.3.4. Methylation Analysis

CPs-1 (5 mg) was methylated using a reported method [32], followed by hydrolyzation and acetylation in sequence. Then, the samples were evaluated by a gas chromatography-mass spectrometry (GC-MS) on a 4000 GC-MS system (Varian, CA, USA). The methodology from the previous literature [33] was employed to analyze the results of methylation.

#### 2.3.5. NMR Spectroscopy Analysis

0.5 mL of D_2_O was mixed with 20 mg of lyophilized CPs-1. A Bruker DRX-400 NMR spectrometer (Rheinstetten, Germany) was used to document ^1^H and ^13^C NMR spectra.

### 2.4. Antioxidant Activity of CPs-1 In Vitro

The radical scavenging abilities and reducing powers were estimated by following the published report [26]. 0.25, 0.5, 1.0, 2.0, and 4.0 mg/mL of different samples were conducted in this study. Vitamin C (VC) was served as the positive control.

### 2.5. Antioxidant Activity of CPs-1 In Vivo

#### 2.5.1. Animal and Treatment

The mouse model generation and treatment of mice were based on previously published method with a slight modification [34]. Kunming mice (KM, male, 20 ± 2 g) were achieved from Si Pei Fu Biotech Co., Ltd. (Beijing, China), which the license number was SCXK (Jing) 2016-0002. Animal studies were conducted following China's Guidelines for the Care and Use of Laboratory Animals. The conduction conforms to the international uses of experimental animals. Mice were maintained in the standard experimental condition (relative humidity: 45-55%, adjustable temperature: 20-25°C), 12 h/12 h light/dark cycle. A normal control group consists of ten healthy KM randomly selected after one-week acclimatization and fed with the basal diet (0.9% normal saline, 0.2 mL/d). The remaining mice were fed with a high-fat diet, composed of 78.8% basic feed, 10% egg yolk powder, 10% lard, 1% cholesterol, and 0.2% sodium cholate, for 30 consecutive days.

After the high-fat mouse model was established, the high-fat mice were randomly grouped into five sections: model control (0.9% normal saline, 0.2 mL/d), positive control (simvastatin, 3.33 mg/kg, 0.2 mL/d), low dose (50 mg/kg, 0.2 mL/d), medium dose (100 mg/kg, 0.2 mL/d), and high dose (200 mg/kg, 0.2 mL/d) groups. Each group of ten mice received continuous gavage for 30 d. The normal control group, throughout the experiment, was fed a basal diet, while the others received ad libitum feeding of a high-fat diet.

When the last 12 h administration was completed, body weight and blood samples through eyeballs of the mice were collected. The serum was obtained by a centrifuge (3000 × *g*) at 4°C for 10 min. The mice were sacrificed through cervical dislocation, and their livers were acquired and weighed. Serum and liver were preserved in the refrigerator at 4°C.

#### 2.5.2. Antioxidant Activity of CPs-1 In Vivo

The indexes of antioxidant, including MDA, CAT, SOD, and GSH-Px, in serum and liver were determined with commercially available kits (Nanjing Jiancheng Bioengineering Institute).

### 2.6. Statistical Analysis

The experimental data were expressed as mean ± standard deviation (SD). The one-way analysis of variance (ANOVA) was conducted using SPSS for Windows version 19.0 (SPSS Inc. Chicago, IL, USA). *P* < 0.05 was used as the cutoff indicating statistical significance.

## 3. Results and Discussion

### 3.1. Purification and Chemical Components of CPs-1

**Figure 1:**
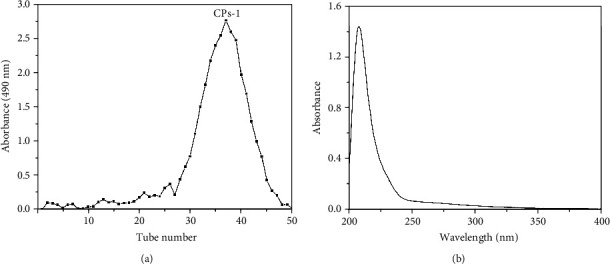
Elution profile of the fraction achieved from deionized water in a gel chromatography column (a) and UV spectrum of CPs-1 (b).

CPs were prepared in a 7.18 ± 0.24% yield from CHMs under optimal conditions based on the reported procedure [26]. Deproteinization and separation of CPs were performed using ultrafiltration centrifugation. As displayed in Figure 1(a), the purified CPs-1 was obtained using the deionized water and Sephadex G-75 column.

The lyophilized CPs-1 was the white powder, with a total carbohydrate content of 97.87%, and no protein or uronic acid was detected, consistent with the results of UV analysis. The UV spectrum of CPs-1 is shown in Figure 1(b). The absence of absorption at 260 and 280 nm implied no protein and nuclear acid in CPs-1, respectively [35].

### 3.2. Molecular Weight Analysis and Composition of Monosaccharide in CPs-1

**Figure 2:**
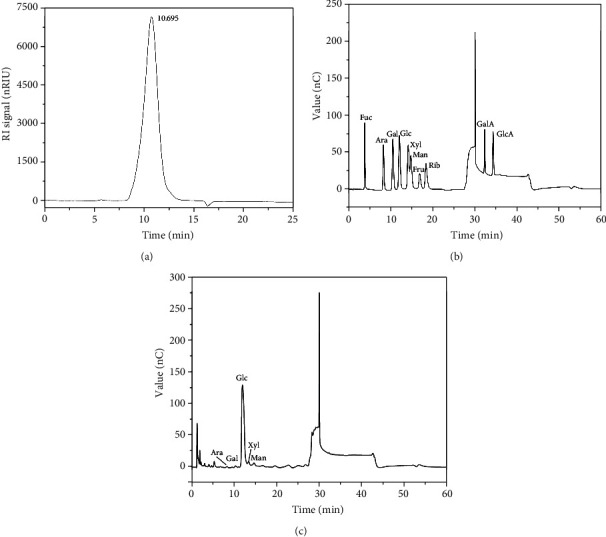
HPGPC of CPs-1 (a) and IC chromatogram of standard monosaccharides (b) and CPs-1 (c).

The homogeneity and average molecular weight of CPs-1 were characterized by HPGPC (Figure 2(a)). The HPGPC profile demonstrated a single symmetrical narrow peak at 10.695 min, suggesting the homogeneity and high purity of CPs-1. The HPGPC analysis revealed that the molecular weight of CPs-1 was 38.7 kDa in terms of the calibration curves of the standard dextrans.

IC was utilized to determine the composition of monosaccharide in CPs-1. Figures 2(b) and 2(c) demonstrated that CPs-1 included Gal, Ara, Xyl, Man, and Glc at a molar ratio of 0.5 : 0.5 : 2.7 : 2.7: 93.6, indicating that CPs-1 were heterogeneous and it is most likely that Glc was the backbone of CPs-1. The result was similar to other reports regarding the MW of polysaccharides extracted from Poria cocos. The composition of CPs-1 in the present work was consistent with that from Semen cassiae at an identical molar ratio of Gal and Glc [36].

### 3.3. FT-IR Analysis of CPs-1

**Figure 3:**
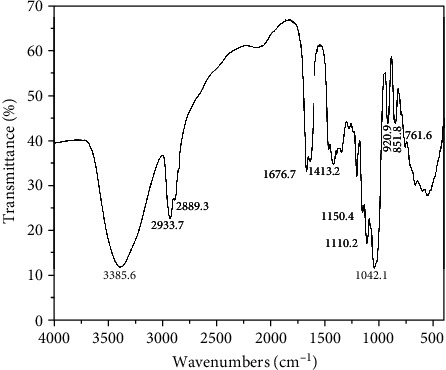
FT-IR spectrum of CPs-1.

The FT-IR spectra were used to analyze the functional groups and chemical bonds of CPs-1 [37]. As shown in Figure 3, the CPs-1 FT-IR spectrogram displayed the typical absorption peaks of polysaccharide, particularly in the regions of 800-1200 cm^−1^, 1400-1700 cm^−1^, 2800-3000 cm^−1^, and 3200-3500 cm^−1^ [26]. The broad and strong peak at approximately 3385 cm^−1^ was attributed to the O-H stretching vibration, associated with the existence of intramolecular and intermolecular hydrogen bonds. The small peaks at approximately 2933 and 2889 cm^−1^ were relevant to C-H stretching trembling from alkyl groups, including CH, CH_2_, and CH_3_ [26, 38]. The absorption peak at around 1676 cm^−1^ could be ascribed to the existence of bound water, while that at approximately 1413 cm^−1^ might be due to the C-H symmetrical deformation vibration [38]. The three absorption bands, i.e., 1150, 1110, and 1042 cm^−1^, in the 1000-1200 cm^−1^ range demonstrated the presence of pyranose ring in the CPs-1 [39]. Moreover, the feature peaks at approximately 920 and 851 cm^−1^ were associated with *β*- and *α*-D-galactopyranose, respectively [40, 41]. The absorption peak at 761.6 cm^−1^ was correlated with *α*-D-xylopyranose [42]. The results were identical to those of monosaccharide composition. In summary, CPs-1 possessed the *α*- and *β*-D-pyranose, in agreement with other polysaccharides from hawthorn and lotus leaf in the previous study [43, 44].

### 3.4. Methylation Analysis of CPs-1

The methylation analysis was executed to evaluate molar ratios and the glycosidic linkage of sugar residues of CPs-1. The main methylated alditol acetates of CPs-1 are estimated to be 2,3,4,6-Me4-D-Glcp, 2,3,4-Me3-D-Glcp, and 2,4-Me2-D-Glcp with a molar ratio of 5.36 : 84.21 : 10.43. The corresponding peak times were 12.92, 16.67, and 19.44 min, respectively. Therefore, the results illustrated the presence of high concentration of the nonreducing terminal glucosyl, (1→6)-linked-glucosyl and (1→3, 6)-linked-glucosyl, and (1→6)-linked-glucosyl in CPs-1. In addition, it was suggested that (1→6)-linked-glucosyl was likely to form the backbone, while (1→3, 6)-linked-glucosyl was identified as the branched residues. The relative molar ratio of (1→6)-linked-glucosyl and (1→3, 6)-linked-glucosyl was 8.07, revealing one branching point for every nine or ten residues of backbone. The nonreducing terminal of 1→linked glucosyl was detected in the branched residues [33].

### 3.5. NMR Analysis of CPs-1

**Figure 4:**
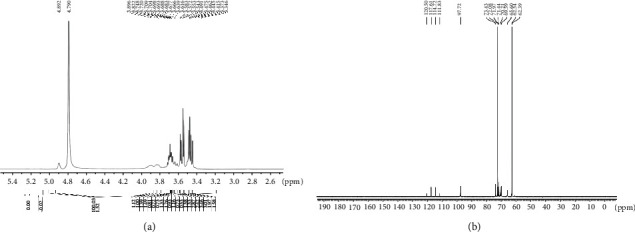
^1^H (a) and ^13^C (b) NMR spectrum of CPs-1.

As indicated in Figures 4(a) and 4(b), the ^1^H and ^13^C NMR spectra of CPs-1 are distributed in a narrow region within *δ*3.0-5.3 ppm (^1^H NMR) and *δ*60-121 ppm (^13^C NMR), correlated with polysaccharides [45]. Based on the composition of the monosaccharide, FT-IR, methylation analysis, and data from literature [33, 46–49], signals in ^1^H and ^13^C NMR spectra of CPs-1 were determined. Because of the overlapped peaks of the interference signal of HDO (*δ*H 4.79 ppm) and the absence of Ara and Gal in the CPs-1, two significantly weak anomeric proton signals occurred at *δ*H 5.0-5.3 ppm, attributed to Ara and Gal. Additionally, other hydrogen on the anomeric carbon with the chemical shift (*δ*H 4.89 ppm) was less than *δ*H 5.0 ppm caused by Glc. This suggested that CPs-1 had both *α*- and *β*-glycosidic bonds. The absences of proton signal peak at *δ*H 5.4 ppm and signal for sugar-ring carbons from *δ*C 82 to 88 ppm (characteristic for furanosides) demonstrated that all sugar residues were pyranose in the CPs-1. The chemical shifts of anomeric carbons at *δ*C 120.50, 117.61, 114.72, 111.83, and 97.72 ppm corresponded to five monosaccharide residues, as well as *α* and *β* anomeric configurations in CPs-1, consistent with ^1^H NMR analysis. The above results were identical with the monosaccharide composition and FT-IR analysis. At the anomeric region of the ^13^C NMR spectrum, signals at 120.50 ppm were allocated to Ara and T-linked *β*-Glcp. Signals at 117.61 and 114.72 ppm were generated from Gal and Glc, respectively, assigned to 1,6-linked *β*-Glcp. Xyl was at 111.83 ppm, originated from 1,3,6-linked *β*-Glcp. Signals at 97.92 ppm were caused by Man. The carbon resonances of CPs-1 from 62.39 to 73.43 ppm were attributed to carbons C2-C6 of various sugar moieties. In general, ^13^C NMR spectrum signals from 67 to 70 ppm validated the existence of (1→6) glycosidic linkages in CPs-1. The methylation analysis and ^1^H and ^13^C NMR spectra of CPs-1 indicated that the presence of (1→6)-*β*-D-Glcp backbone and O-3 position of (1→6)-linked *β*-D-Glcp was substituted by 1-linked *β*-D-Glcp branches, with one branching point for every 15 or 16 backbones on average.

### 3.6. Antioxidant Activities of CPs-1 In Vitro

DPPH has been extensively used to measure the oxidation resistance of various antioxidants, characterized by simple, rapid, and sensitive properties. The ABTS radical scavenging is also broadly applied to evaluate the total antioxidant capacity of polysaccharides. Removing hydroxyl radicals and superoxide anion will protect the body against oxidization-related damage. The strong reducing power indicates high antioxidant activities [26].

#### 3.6.1. DPPH Radical Scavenging Activity

**Figure 5:**
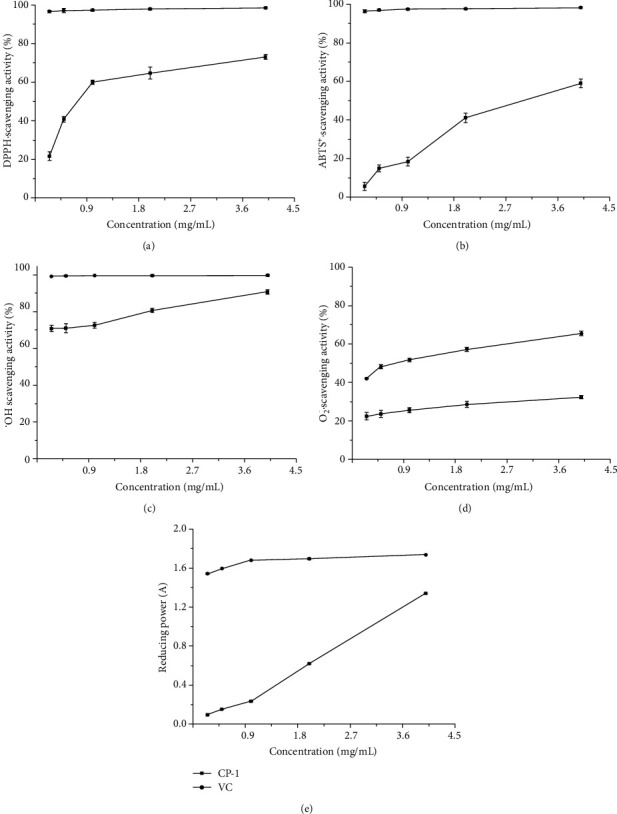
Antioxidant activities of CPs-1 for DPPH scavenging capacity compared to that of VC (a), for ABTS scavenging capacity compared to that of VC (b), for hydroxyl radical scavenging capacity compared to that of VC (c), for superoxide radical scavenging capacity compared to that of VC (d), and for reducing power compared to that of VC (e).

Figure 5(a) presents the scavenging ability of CPs-1 for DPPH compared to that of VC. It can be observed that the scavenging capacity increases significantly with the polysaccharides from 0.25 to 4.0 mg/mL, inferior to that of VC at all concentrations. The scavenging activity of CPs-1 and VC at 4.0 mg/mL is 73.09 ± 1.35% and 98.47 ± 0.51%, respectively. The scavenging ability of CPs-1 at 2.0 mg/mL is higher than that of hawthorn polysaccharides (HTPs) and Lycium barbarum polysaccharides (LBPs) in previous research [43, 50]. Thus, CPs displayed noteworthy DPPH radical scavenging activity because an electron or a hydrogen atom could be easily provided.

#### 3.6.2. ABTS Radical Scavenging Activity

CPs-1 displayed the scavenging capacity against ABTS, highly depending on the polysaccharide concentration of 0.25 to 4.0 mg/mL (Figure 5(b)). It was indicated that the ABTS scavenging ability is weaker than that of VC. At a concentration of polysaccharide (4.0 mg/mL), the scavenging activity is 58.93 ± 2.17%, significantly lower than that of VC (98.13 ± 0.34%). The conclusion is in agreement with other published reports in the scavenging capacities for ABTS of HTPs and Fagopyrum tataricum polysaccharides (FTPs) [51, 52]. In short, CPs exhibited great scavenging ability for ABTS, enabling it potentially to be used as a novel antioxidant from natural resources.

#### 3.6.3. Hydroxyl Radical Scavenging Activity

As demonstrated in Figure 5(c), the hydroxyl radical scavenging ability of CPs-1 is greatly correlated with the polysaccharides concentrations between 0.25 and 4.0 mg/mL. The scavenging capacity is 90.75 ± 1.15%, lower than that of VC (99.79 ± 0.42%) at 4.0 mg/mL. The scavenging activity of CPs-1 for hydroxyl radical exceeds those in LBPs and Poria cocos polysaccharides (PCPs) reported in other publications [18, 53]. It is due to the fact that the hydroxyl radicals were scavenged by polysaccharides interacting with hydrogen with radicals and discontinued the radical chain reaction.

#### 3.6.4. Superoxide Radical Scavenging Activity

As illustrated in Figure 5(d), the scavenging capacity of CPs-1 at 4.0 mg/mL for superoxide radical is 32.31 ± 0.79%, compared with 65.54 ± 1.17% of VC. The superoxide radical scavenging abilities of CPs-1 are lower than those of the results from previous studies on HTPs, FTPs, LBPs, and Semen cassiae polysaccharides (SCPs) [16, 51, 53, 54]. To sum up, CPs-1 has relatively weak ability of scavenging the superoxide radical.

#### 3.6.5. Reducing Power

The comparison of the absorbance between CPs-1 and VC is demonstrated in Figure 5(e). The absorbance of CPs-1 (4.0 mg/mL) and VC is 1.342 ± 0.002 and 1.738 ± 0.002, respectively. The reducing power of CPs-1 is stronger than those of PCPs and LBPs in literature [18, 53].

The highly correlated relationship among the radical scavenging abilities (ABTS, hydroxyl, and superoxide anion), reducing powers, and CPs-1 concentration is described in Table 1, with correlation coefficients (*R*^2^) of 0.9571, 0.9861, 0.9716, and 0.9923, respectively. In addition, a linear relationship (*R*^2^ = 0.9369) was observed between the DPPH scavenging capacity and CPs-1 concentration.

### 3.7. Antioxidant Activities of CPs-1 In Vivo

SOD is specialized in scavenging superoxide anions and can improve the immunity and resistance of the body to ROS-induced diseases. As one of terminal oxidases, CAT is able to decompose hydrogen peroxide in cells, preventing tissue damage [55]. GSH-Px can reduce the free hydrogen peroxide to water and toxic lipid hydroperoxides preventing cells from ROS toxicity [56]. Therefore, CAT, GSH-Px, and SOD are the most crucial indexes to assess the antioxidant capacity. The MDA level reflects the oxidative damage severity and lipid peroxidation level *in vivo* [2].

#### 3.7.1. Contents of CAT, GSH-Px, MDA, and SOD in Serum of Mice

Table 2 illustrates the effects of CPs-1 on serum concentrations of CAT, SOD, and GSH-Px, reflecting their remarkable increases in serum. Moreover, the MDA level in serum decreases gradually with the increase of the dosage of CPs-1. It was proved that the impacts of high-dosage CPs-1 on CAT, GSH-Px, and SOD concentrations in serum were substantially different from those of the model control (*P* < 0.01) and normal control (*P* < 0.01) groups, indicating significant differences in the influences of the medium-dose and the model control group on the concentrations of CAT, GSH-Px, and SOD in serum. In comparison with the model control group, the concentrations of MDA in serum with high- and medium-dosages of CPs-1 were substantially lower, where the significance level was 0.01 and 0.05, respectively. Compared with the normal control group, the contents of CAT, GSH-Px, and SOD in serum increased by 32.72%, 56.17%, and 87.24% in sequence with high-dosage CPs-1 (*P* < 0.01). These demonstrated that CPs-1 could dramatically reduce the concentration of MDA in serum since no significant difference was identified between these two groups (*P* > 0.05).

#### 3.7.2. Concentrations of CAT, GSH-Px, MDA, and SOD in the Liver of Mice

The determinations of CAT, GSH-Px, MDA, and SOD in the liver of mice are displayed in Table 3. As shown in Table 3, the concentrations of CAT, GSH-Px, and SOD were substantially increased, while that of MDA was reduced gradually with increasing dosage of CPs-1, demonstrating the differential effects of high-dosage CPs-1 and the model control group (*P* < 0.01) on the concentrations of CAT, GSH-Px, MDA, and SOD in the liver. The concentrations of SOD in the liver with high- and medium-dosage CPs-1 were evidently higher than those of the model control group, where the significance levels were 0.01 and 0.05, respectively. Compared with that of the normal control group, the concentrations of CAT, GSH-Px, and SOD in the liver of mice in the high-dosage group were increased by 17.39% (*P* < 0.05), 81.79% (*P* < 0.01), and 9.77%, respectively. Therefore, CPs-1 could prominently diminish MDA concentration in the liver of mice.

## 4. Conclusions

The CPs-1 were successfully purified from crude CPs by column chromatography, with an average molecular weight of 38.7 kDa. CPs-1 were heterogeneous polysaccharides, consisting of Gal, Ara, Xyl, Man, and Glc at a molar ratio of 0.5 : 0.5 : 2.7 : 2.7 : 93.6. In addition, the FT-IR spectra exhibited the presence of the *α*- and *β*-D-pyranose in CPs-1. The methylation analysis and NMR spectrum indicated that CPs-1 had a →6)-*β*-D-Glcp-(1→6)-*β*-D-Glcp(1→ backbone. The results of antioxidant activities *in vitro* suggested that CPs-1 showed high scavenging abilities for DPPH (73.09 ± 1.35%), hydroxyl (90.75 ± 1.15%), and reducing power (1.342 ± 0.002) at 4.0 mg/mL, as well as ABTS and superoxide scavenging capacity. Furthermore, CPs-1 could significantly enhance enzymatic activities (i.e., CAT, GSH-Px, and SOD) and distinctly reduce MDA serum- and liver-concentrations in high-fat mice. Hence, CPs-1 could function as a natural novel antioxidant for medicine and functional foods. Further research is currently performed to investigate the antioxidation mechanism of CPs-1.

## Figures and Tables

**Table 1:** The correlation between antioxidant activity and concentration of CPs.

Antioxidant arrays	Optimal fitting functions	Determination coefficients (*R*^2^)
DPPH	*Y*_*a*_ = 18.28Ln(*X*_*c*_) + 52.04	0.9369
ABTS	*Y*_*a*_ = 13.959*X*_*c*_ + 6.1463	0.9571
Hydroxyl	*Y*_*a*_ = 5.609*X*_*c*_ + 68.472	0.9861
Superoxide-anion	*Y*_*a*_ = 2.576*X*_*c*_ + 22.499	0.9716
Reducing power	*Y*_*a*_ = 0.3392*X*_*c*_ − 0.0375	0.9923

*X_c_* was the concentration of CPs, and *Y_a_* was the antioxidant activity corresponding to *X_c_*.

**Table 2:** Quantification of serum level CAT, GSH-Px, MDA, and SOD.

Groups	CAT contents (U/mL)	GSH-Px contents (U/mL)	MDA contents (nmol/mL)	SOD contents (U/mL)
Normal control	13.60 ± 1.01^a^	664.23 ± 45.96^b^	3.32 ± 0.35^a^	100.12 ± 10.47^a^
Model control	10.80 ± 0.70^∗^	460.86 ± 43.37^∗∗^	5.14 ± 0.78^∗^	59.64 ± 19.68^∗^
Low dose	12.36 ± 1.09	809.07 ± 107.74^b∗^	4.08 ± 0.91	79.53 ± 19.40^a^
Medium dose	15.32 ± 1.28^b^	924.65 ± 58.13^b^^∗∗^	3.53 ± 0.78^a^	142.01 ± 10.88^b^^∗^
High dose	18.05 ± 1.30 b^∗∗^	1037.31 ± 49.51^b^^∗∗^	3.02 ± 0.85^b^	187.46 ± 25.19^b^^∗∗^

^a^Statistical significance level: *P* < 0.05 (vs. the model control group). ^b^Statistical significance level: *P* < 0.01 (vs. the model control group).^∗^Statistical significance level: *P* < 0.05 (vs. normal control group). ^∗∗^Statistical significance level: *P* < 0.01 (vs. normal control group).

**Table 3:** Quantification of liver level CAT, GSH-Px, MDA, and SOD.

Groups	CAT contents (U/mgprot)	GSH-Px contents (U/mgprot)	MDA contents (nmol/mgprot)	SOD contents (U/mgprot)
Normal control	68.02 ± 5.07^b^	832.90 ± 77.41^a^	6.80 ± 0.58^b^	287.92 ± 23.14^a^
Model control	54.02 ± 3.48^∗∗^	655.53 ± 36.96^∗^	10.73 ± 1.03^∗∗^	250.16 ± 8.76^∗^
Low dose	61.79 ± 5.44	925.45 ± 62.27^b^	7.70 ± 0.58^b^	267.34 ± 8.24
Medium dose	74.34 ± 4.69^b^	1213.37 ± 156.62^b^^∗∗^	6.85 ± 0.99^b^	283.66 ± 14.19^a^
High dose	79.85 ± 7.13^b^^∗^	1514.14 ± 94.76^b^^∗∗^	6.19 ± 0.76^b^	316.06 ± 24.73^b^

^a^Statistical significance level: *P* < 0.05 (vs. the model control group). ^b^Statistical significance level: *P* < 0.01 (vs. the model control group).^∗^Statistical significance level: *P* < 0.05 (vs. normal control group).^∗∗^Statistical significance level: *P* < 0.01 (vs. normal control group).

## Data Availability

Data is contained within the article.
